# Editorial: Hypertension: Novel Mechanisms of Nervous and Humoral Regulation

**DOI:** 10.3389/fphar.2022.937673

**Published:** 2022-06-15

**Authors:** Peng Li, Haijian Sun, Haiyang Tang

**Affiliations:** ^1^ Department of Cardiology, The First Affiliated Hospital of Nanjing Medical University, Nanjing, China; ^2^ Department of Pharmacology, National University of Singapore, Singapore, Singapore; ^3^ State Key Laboratory of Respiratory Disease, Guangdong Key Laboratory of Vascular Disease, National Clinical Research Center for Respiratory Disease, Guangzhou Institute of Respiratory Health, The First Affiliated Hospital of Guangzhou Medical University, Guangzhou, China

**Keywords:** Hypertension, Vascuar function, pulmonary hypertension, sympathetic activation, cardiovascukar diseases

The growing burden of hypertension is emerging as one of the major healthcare challenges in low- and middle-income countries. Uncontrolled hypertension places a high burden on both patients and healthcare providers. Though several treatments available such as some of anti-hypertensive medication. The most effective treatments for hypertension and hypertension-associated cardiovascular ailment are still lacking. In this Research Topic, we collected articles to increase the understanding behind the mechanisms of humoral and nervous regulation on hypertension, and explore novel therapeutic targets to combat hypertension and associated cardiovascular diseases. Research Topics of interest include, but are not limited to the mechanism of sympathetic and parasympathetic nerves on the regulation of hypertension, signaling pathways involved in the progression of hypertension and cardiovascular diseases, G protein-coupled receptors in hypertension and cardiovascular diseases, hypertension in pregnancy, and pulmonary hypertension treatment. After the joint efforts of the journal, editors, reviewers, and contributors, a total of six high-quality articles were received. Although small in the kingdom of hypertension research, these six articles have certainly contributed to advancing understanding of hypertension pathogenesis and interventions. We have made a detailed summary and perspective for these six articles as follows.

Endoplasmic reticulum (ER) stress potentiates neuroinflammation and neurodegeneration and is a key contributor to the pathogenesis of neurogenic hypertension. Kinin B1 receptor (B1R) activation plays a vital role in modulating neuroinflammation and hypertension. With regard to this, White et al. examined whether B1R activation results in the progression and enhancement of ER stress. They found that B1R stimulation induced the upregulation of GRP78, a molecular chaperone of ER stress in association with an increased expression and activation of transmembrane ER stress sensors, ATF6, IRE1α, and PERK, the critical components of UPR. In the presence of overwhelming ER stress, activated ER stress sensors can lead to oxidative stress, autophagy, or apoptosis. Thus, B1R activation initiates the UPR and is a key factor in the ER stress pathway, which is implicated in the pathologies of neurogenic hypertension. Music has been well known to elicit emotional changes, such as anxiolytic effects. However, whether music therapy lowers BP in spontaneously hypertensive rats (SHR) and the potential mechanism remains unknown. Li J. et al. uncovered that Chinese classical music lowered systemic blood pressure and alleviated myocardial hypertrophy in hypertensive rats, which may be caused by the inhibitions of β1/cAMP/PKA and α1/PLC/PKC signaling. Thereby, the results of our study suggest that Chinese classical music therapy may be an alternative and adjuvant therapy for hypertension, which has important academic value and bright clinical application prospect. Magnolia volatile oil (MVO) is a mixture mainly containing eudesmol and its isomers. Xu et al. investigated the vasorelaxant effects and the underlying mechanism of MVO in rat thoracic aortas. MVO exerted a vasorelaxant effect on the aortic rings pre-contracted by KCl and phenylephrine in an endothelium-dependent and non-endothelium-dependent manner. Additionally, MVO could significantly inhibit Ca^2+^ influx resulting in the contraction of aortic rings, revealing that the inhibition of the calcium signaling pathway exactly participated in the vasorelaxant activity of MVO as predicted by network pharmacology. The network pharmacology research predicted that beta-caryophyllene, palmitic acid, and (+)-β-selinene might act as the effective ingredients of MVO for the treatment of hypertension. As a consequence, MVO might be a potent treatment of diseases with vascular dysfunction like hypertension. Li Y.et al. retrieved relevant literature using PubMed database until 30 August 2021 to clarify the association between antihypertensive use and risk of depression. They found that the usage of angiotensin antagonists, beta blockers and calcium channel blockers are potential risk factors of depression.

Pregnancy with pulmonary hypertension (PH) seriously threatens the life and safety of mothers and infants. The long-term effect of maternal PH on the postpartum growth of rat offspring receives enormous attention to date. Wang et al. revealed that targeting Myadm to intervene PH before pregnancy could alleviate sustained low weight growth in maternal PH rat offspring, and the pathological changes of the cardiac–cerebral system. Sodium-glucose cotransporter-2 (SGLT2) inhibitors, a novel class of hypoglycemic drugs, show excellent cardiovascular benefits, and have further improved heart failure outcomes, significantly reducing cardiovascular and all-cause mortality irrespective of diabetes status. Unfortunately, a study by Li H. et al. found that dapagliflozin treatment did not significantly improve experimental pulmonary arterial hypertension and pulmonary trunk banding in rat PAH models induced by monocrotaline (MCT). This is a negative therapeutic experiment, suggesting human trials with dapagliflozin for PAH or RV failure should be cautious. Totally, these six articles collected in this Research Topic highlighted the recent updates of hypertension prevention and treatment to some extent from different viewpoints. Eventually, we look forward to seeing more interesting research in this area, and highlighting clinical perspectives to combat adverse high blood pressure and hypertension-associated cardiovascular diseases.

